# Commentary: Pride, Shame, and Group Identification

**DOI:** 10.3389/fpsyg.2016.01946

**Published:** 2016-12-15

**Authors:** Mikko Salmela, Gavin Brent Sullivan

**Affiliations:** ^1^Department of Political and Economic Studies/Social and Moral Philosophy, University of HelsinkiHelsinki, Finland; ^2^Centre for Research in Psychology, Behaviour and Achievement, Coventry UniversityCoventry, UK

**Keywords:** appropriateness, size, shape, hetero-induced self-conscious emotions, group-based emotion

In the target article, Alessandro Salice and Alba Montes Sánchez argue that people sometimes feel proud and ashamed of the actions of others whom they perceive to belong to the same group as themselves. The authors maintain that the social self is the target of emotion whereas the other is merely its focus—a background object that makes intelligible the target's instantiating the formal object of the emotion type.

We accept the authors' phenomenological analysis of the intentional structure of hetero-induced shame and pride. Instead, we raise a question about the appropriateness of these emotions that the authors address only briefly by claiming that hetero-induced pride and shame are appropriate in some cases, without specifying what these cases might be. Conventional emotion norms provide one guideline, but they are not consistent. For instance, parents' and grandparents' pride in the success of their offspring is a warranted emotion in American family ideology, perhaps elsewhere in the world as well. Yet children's shame of the drunken or criminal behavior of their parents is regarded as an inappropriate emotion from which children should emancipate themselves. These examples show that we need a more systematic approach to the appropriateness of hetero-induced pride and shame.

An important distinction in appropriateness concerns the shape and size of an emotion (D'Arms and Jacobson, 2000). An emotion is appropriate in terms of shape if its particular object has properties that render it an instance of the formal object of the emotion type, whereas it is appropriate in terms of size when the emotional response is neither too intense nor too mild, both in feeling and display. But how to cash out these criteria for hetero-induced pride and shame?

Richard Lazarus defines the formal object or core relational theme of pride as “enhancement of one's ego-identity by taking credit of a valued object or achievement, either of our own or that of someone or group with whom we identify” (Lazarus, 1991, 122). Interestingly, this definition already involves the case of hetero-induced pride, unlike shame that according to Lazarus is felt about failure to live up to one's ego-ideal. Yet we believe that it is possible to give appropriateness conditions for both hetero-induced pride and shame.

In group contexts, it is one thing to celebrate the achievement of one's fellow group members, thereby expressing one's membership, and another thing to feel proud of oneself by virtue of such achievement. The former does not imply the latter, and even if people use such expressions as “I am proud of what you did,” they are not typically taking credit of the actions of others and feeling proud of themselves, but rather praising those others and their actions (Sullivan, 2007). In some group contexts where people feel proud of the achievements of others, the emotion can be felt as sharing in our pride of something that we did together. Here the self is involved in the “we” that is collectively committed to achieving its goal, and thereby every group member can take credit and feel proud of the joint achievement.

Still, the question remains how to apply this account to friends and families that cannot be understood as we-mode groups (Tuomela, 2013) in a straightforward sense. Nor is there an experience of sharing in our pride when for instance parents feel proud of themselves by virtue of their daughter's achievement.

One consideration here is to what extent hetero-induced pride and shame are empathic responses to the emotions of others. We readily empathize with our significant others and share the emotions that we perceive or imagine them feeling. However, empathically shared emotions should not be interpreted as hetero-induced emotions of my social self in the first place.

Second, causal contribution to the success or failure of the other may be important for the appropriateness of hetero-induced pride and shame even if it does not figure in the phenomenology of these emotions. The example of proud parents is a case in point. It may be appropriate in terms of shape only in cases where the parents with their caring and loving parenting have contributed to the success of their offspring. Accordingly, foster parents can feel appropriately proud of the success of their adopted children, whereas there is something repulsive in the pride of biological parents for the achievements of children who have been taken into custody and raised by others because of the parents' neglectful or abusive behavior. We can see some appropriateness in the pride of such parents if they regret their early abuse, yet not as much as in the pride of proper biological or foster parents.

Finally, there is the dimension of size in appropriateness. This is a problem with parents and grandparents who boast about the achievements of their offspring as this kind of flamboyant expression betrays inappropriate intensity of the emotion. Such parents or grandparents use their offspring as assets in social rivalry, which is morally repulsive because human beings should not be treated as means but as ends in themselves, to use Kant's expression. In general, subjects of hetero-induced pride or shame should tone down the intensity of their emotion, recognizing the origin of the emotion in the actions of others who are justified to express the emotion more intensely.

The last point is salient in the case of parents and grandparents who feel proud of the achievements of their offspring. However, it is less salient in the case of fans who celebrate the success of their favorite team in spectator stands or public viewing areas where shared emotions also serve the purpose of reinforcing the participants' collective identity and mutual solidarity. In such situations, ritualistic sharing of emotions intensifies them into collective effervescence as Durkheim (1912/2001) already observed. The fact that other factors and motives influence emotion experience and expression in collective contexts renders those situations more complicated than the case of parents. Indeed, shared group-based pride should not be conceptualized as hetero-induced personal pride.

## Author contributions

MS is the first author who drafted the article and GS the second author who provided extensive comments on it.

## Funding

The first author's research is funded by the Academy of Finland Centre of Excellence in the Philosophy of Social Sciences, decision number 284631.

### Conflict of interest statement

The authors declare that the research was conducted in the absence of any commercial or financial relationships that could be construed as a potential conflict of interest.
